# Supplementary material for: Subunit Vaccine Targeting Phosphate ABC Transporter ATP-Binding Protein, PstB, Provides Cross-Protection against Streptococcus suis Serotype 2, 7, and 9 in Mice
Source: Vet Sci. 2023 Jan 9;10(1):48. doi: 10.3390/vetsci10010048 (PMC9953333; doi:10.3390/vetsci10010048)

Figure S1 Bioinformatic analysis of PstB

(A) Hydrophobicity analysis. The hydrophobicity analysis was performed using software ProtScale (https://web.expasy.org/protscale/).

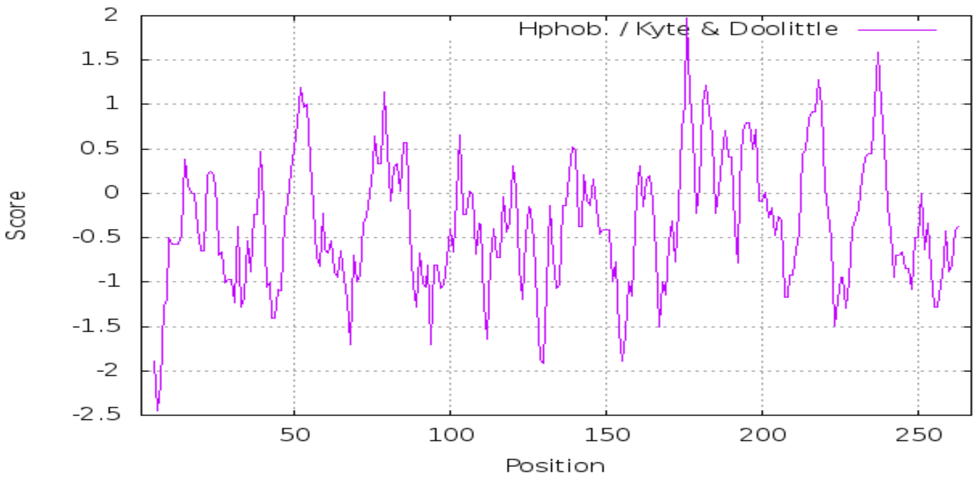

(B) Transmembrane domain analysis. The transmembrane domain was analyzed using the software THMHH (http://www.cbs.dtu.dk/services/TMHMM/).

```
# WEBSEQUENCE Length: 267
# WEBSEQUENCE Number of predicted TMHs:  0
# WEBSEQUENCE Exp number of AAs in TMHs: 0.00635
# WEBSEQUENCE Exp number, first 60 AAs:  0.00262
# WEBSEQUENCE Total prob of N-in:        0.15608
WEBSEQUENCE     TMHMM2.0        outside      1   267
```

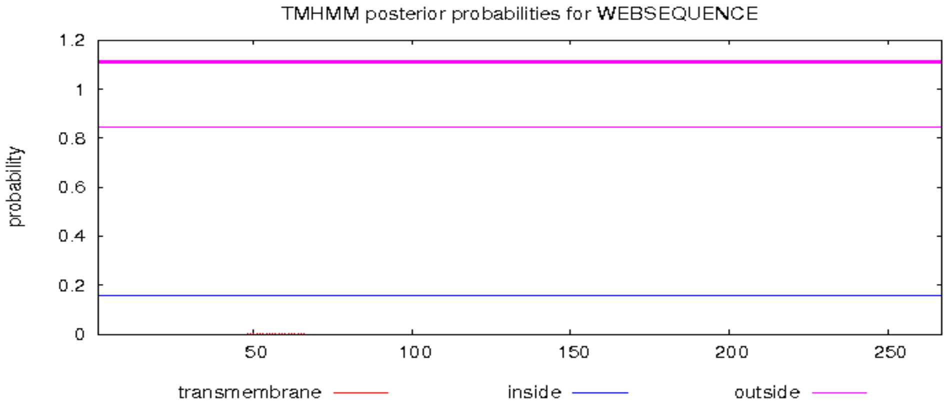

(C) Antigenicity analysis. The antigenicity was analyzed using Protean software of DNAStar package.

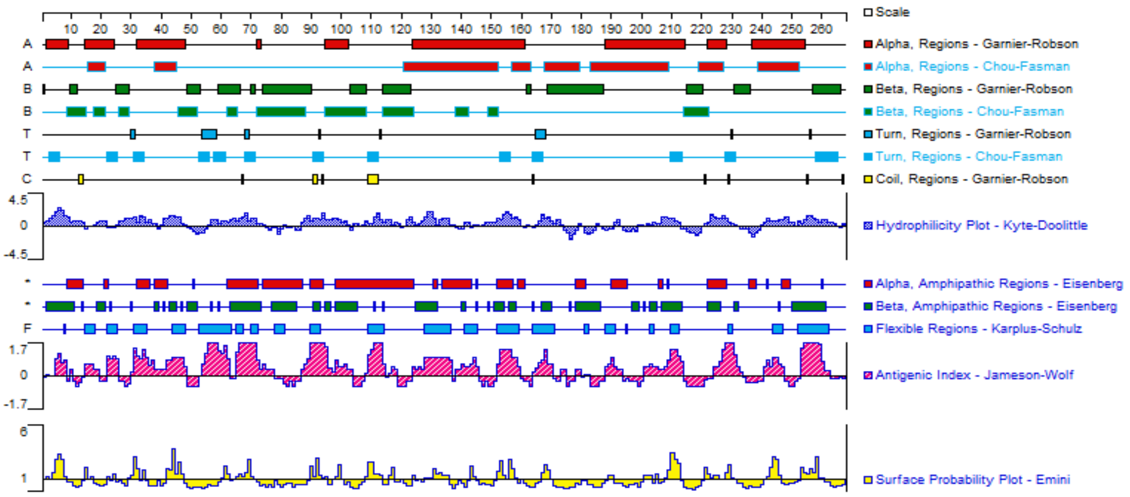

Supplement: Supplementary file 1 [file vetsci-10-00048-s001.zip › Figure S1 Bioinformatic analysis of PstB.pdf]
