# Supplementary figures and images for: Subunit Vaccine Targeting Phosphate ABC Transporter ATP-Binding Protein, PstB, Provides Cross-Protection against Streptococcus suis Serotype 2, 7, and 9 in Mice
Source: Vet Sci. 2023 Jan 9;10(1):48. doi: 10.3390/vetsci10010048 (PMC9953333; doi:10.3390/vetsci10010048)

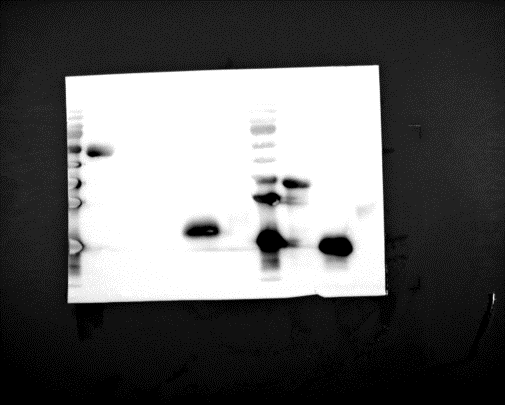

Supplement: Supplementary file 1 [file vetsci-10-00048-s001.zip › Figure S2 The original western blot of Figure 3C-left.png]

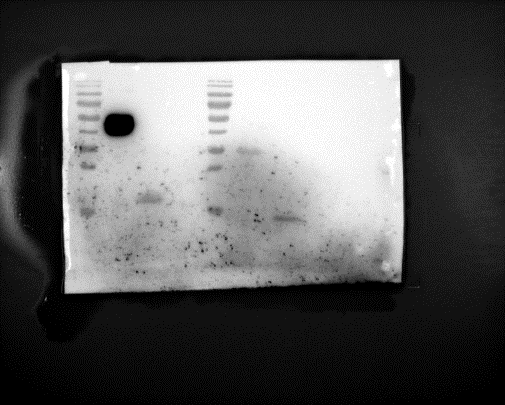

Supplement: Supplementary file 1 [file vetsci-10-00048-s001.zip › Figure S3 The original western blot of Figure 3C-right.png]
